# Isolation of *Arthrobacter* species from the phyllosphere and demonstration of their epiphytic fitness

**DOI:** 10.1002/mbo3.59

**Published:** 2013-01-28

**Authors:** Tanja R Scheublin, Johan H J Leveau

**Affiliations:** 1 Department of Microbial Ecology, Netherlands Institute of Ecology (NIOO-KNAW)Droevendaalsesteeg 10, Wageningen, 6708 PB, The Netherlands; 2 Department of Plant Pathology, University of CaliforniaOne Shields Avenue, Davis, California, 95616

**Keywords:** *Arthrobacter*, biodegradation, phylloplane, phylloremediation, soil, triadimenol

## Abstract

Bacteria of the genus *Arthrobacter* are common inhabitants of the soil environment, but can also be recovered from leaf surfaces (the phyllosphere). Using enrichment cultures on 4-chlorophenol, we succeeded in specifically isolating *Arthrobacter* bacteria from ground cover vegetation in an apple orchard. Based on 16S rRNA gene sequencing, the isolates were found to belong to at least three different species of *Arthrobacter*. Compared to the model bacterial epiphyte *Pantoea agglomerans*, the *Arthrobacter* isolates performed as well or even better in a standardized laboratory test of phyllosphere fitness. A similar performance was observed with the well-characterized soil isolate *Arthrobacter chlorophenolicus* A6. These findings suggest that the frequently reported presence of *Arthrobacter* strains on plant foliage can be explained by the capacity to multiply and persist in the phyllosphere environment. As bacteria from the genus *Arthrobacter* are known for their ability to degrade a wide variety of organic pollutants, their high phyllosphere competency marks them as a promising group for future studies on phyllosphere-based bioremediation, for example, as foliar bioaugmentation on ground cover or buffer-zone vegetation to prevent pesticides from reaching soil, surface-, or groundwater.

## Introduction

The phyllosphere (Ruinen 1961) is an open habitat that harbors large and diverse communities of bacteria, fungi, and other microorganisms (Leveau 2006). One of the bacterial genera that show up frequently in culture-independent surveys of leaf surface microbiota is *Arthrobacter* (high %GC Gram-positive, family Micrococcaceae, order Actinomycetales, phylum Actinobacteria). For example, on the leaves of harvest-ready lettuce plants, *Arthrobacter* sequences were found consistently across samples (Rastogi et al. 2012). *Arthrobacter* strains have also been isolated from leaves of strawberry (Krimm et al. 2005), sugar beet (Thompson et al. 1995), potato (Heuer and Smalla 1999), the resurrection fern *Polypodium polypodioides* (Jackson et al. 2006), and olive trees (Ercolani 1991).

Thus, the presence of *Arthrobacter* on leaf surfaces is an established aspect of phyllosphere microbiology. We are interested in the drivers that underlie this presence, which evokes the basic question whether bacteria from the genus *Arthrobacter* constitute so-called residual or transient epiphytes (Whipps et al. 2008). Classical examples of residuals are representatives of the genera *Pseudomonas*, *Pantoea,* and *Erwinia*: they are defined (Whipps et al. 2008) by the capacity to multiply in the phyllosphere (Manulis et al. 1998; Mercier and Lindow 2000; Sabaratnam and Beattie 2003). By contrast, transients lack this capacity. For example, *Bacillus* species have been shown to be poor leaf colonizers even under conducive laboratory conditions (Maduell et al. 2008).

Given their ability to multiply, one expects residual epiphytes to be more abundantly represented than transients in the bacterial communities on plant foliage. Indeed, for genera such as *Pseudomonas*, *Pantoea*, and *Erwinia,* this tends to hold true (Leveau and Tech 2011; Yashiro et al. 2011; Rastogi et al. 2012). Lacking the ability to produce offspring, transients are more likely to be part of the “rare biosphere” component (Kunin et al. 2010) of bacterial communities on plant leaves. This, however, is not a general rule. For example, bacteria of the genus *Bacillus* can constitute a significant portion of the leaf microbiota (Leveau and Tech 2011). This can be explained by assuming high immigration rates of these bacteria to the leaf surface from other sources, rather than multiplication on the leaf surface (Maduell et al. 2008).

Immigration from soil represents one likely mechanism to explain the presence of *Arthrobacter* on leaf surfaces of plants. *Arthrobacter* species are abundant in soil (Mongodin et al. 2006) and soil particles are common on foliage of plants that are grown outdoors (Monier and Lindow 2004). Wind and rain splatter may deliver soil particles to leaf surfaces, especially if the leaves are close to the soil line. In a study that compared bacterial diversity of the lettuce phyllosphere to that of the soil in which these plants were grown, it was revealed that many bacterial species were common between the two compartments (Zwielehner et al. 2008). This was taken as indirect evidence for the movement of soil bacteria to the lettuce canopy. The transport of bacteria by soil particles across larger spatial scales has also been documented (Hua et al. 2007; Polymenakou et al. 2008).

A second contributing factor to the foliar presence of *Arthrobacter* would be the capacity of *Arthrobacter* to multiply in the phyllosphere. To the best of our knowledge, a test of such capacity, that is a test of *Arthrobacter*'s residual nature, has not yet been reported. Demonstration of high epiphytic fitness for *Arthrobacter* would constitute an important finding toward the broader and longer term goal of elucidating the assembly rules that shape phyllosphere communities (Meyer and Leveau 2012).

A particularly interesting property of *Arthrobacter* species is that they can degrade a wide variety of organic pollutants. These include aromatic hydrocarbons, such as phenols, chlorophenols, BTEX compounds, and phenanthrene (Alvarez and Vogel 1991; Keuth and Rehm 1991; Westerberg et al. 2000; Kotouckova et al. 2004), s-triazines such as atrazine and cyanazine, phenylurea herbicides, glyphosate, and malathion (Kertesz et al. 1994; Strong et al. 2002; Tixier et al. 2002). Nicotine-degrading *Arthrobacter* strains have been isolated from the tobacco phyllosphere (Sguros 1955) and oil-utilizing *Arthrobacter* bacteria were isolated from the phyllosphere of crops grown on oil-contaminated soil (Al-Awadhi et al. 2009).

We report here the targeted isolation of *Arthrobacter* strains from leaf surfaces by exploitation of the fact that *Arthrobacter* species can grow at the expense of aromatic pollutants including 4-chlorophenol (4-CP). We used 4-CP enrichment cultures to isolate *Arthrobacter* strains from plant leaves in an apple orchard and we confirmed their epiphytic fitness in laboratory tests. We discuss our findings in the context of exploiting culturable *Arthrobacter* strains for phylloremediation (Sandhu et al. 2007), that is the removal of foliage-associated organic pollutants by members of the phyllosphere community.

## Materials and Methods

### Sampling

Epiphytic bacteria were recovered from foliage at an experimental apple orchard (Applied Plant Research or PPO, Randwijk, The Netherlands), which had received weekly treatments with the foliar fungicide triadimenol (Exact®; Bayer CropScience B.V., Monheim, Germany). One of the main photodegradation products of triadimenol is 4-CP (Wang and Lemley 2003; Da Silva and Vieira Ferreira 2004). From each one of six plots (A–F), a composite sample consisting of 16 apple leaves and a composite sample consisting of ground cover (i.e., grass and herb vegetation dominated by *Poa pratensis*, common meadow grass; *Poa annua*, annual meadow grass; *Stellaria media*, common chickweed; *Senecio vulgaris*, common groundsel) was weighed at 10.9 ± 1.0 and 7.3 ± 1.3 g (average ± standard deviation), respectively, and washed in 100 mL phosphate-buffered saline (PBS) by vortexing (5 sec), sonication (7 min), and vortexing again (5 sec). Leaf washes were concentrated 17-fold by centrifugation and resuspended in PBS, and 0.75-mL aliquots were used to inoculate 15 mL Brunner mineral medium (MM; DSMZ medium no. 457, Braunschweig, Germany) containing 1 mmol/L 4-CP or 0.3 mmol/L triadimenol (Sigma-Aldrich, Zwijndrecht, The Netherlands). Media to which no bacteria were added served as controls. Bottles were incubated at 25°C while shaking at 150 rpm for 4 weeks. Every week, 1 mL of culture was collected and frozen at −20°C for high-performance liquid chromatography (HPLC) analysis of 4-CP and triadimenol.

### HPLC measurements

Frozen samples were thawed, filtered over a 0.2-μm filter, and analyzed by HPLC. We used an ASI-100/ASI-100T Autosampler, STH 585 Column Thermostat, UVD 170U/340U UV/VIS Detector, and P680 LPG pump (Dionex, München, Germany). The UV detector was set at 227 nm for 4-CP and at 224 nm for triadimenol. Runs were performed on a reverse phase C-18 column, 3 μm, 150 × 4.6 mm (Grace Davison Discovery Science, Deerfield, IL) at a column temperature of 25°C and a flow rate of 1 mL per min with 50% acetonitrile as the eluent. The injection volume was 25 μL for the 4-CP and 50 μL for the triadimenol samples.

### Isolation of bacteria

After 2 weeks of enrichment, serial dilutions of the 4-CP cultures that were inoculated with bacteria from the grass–herb mixture were spread on 1/10 Tryptone Soy Agar (TSA; Oxoid, Cambridge, UK) with 15 g agar per liter. For each one of the six cultures, 12–16 single colonies were transferred to fresh TSA plates and restreaked twice for purity. Care was taken to include a representative from each morphologically distinct colony type. Each isolated strain was checked for its ability to grow in MM with 1 mmol/L 4-CP. Sixteen of those that did were selected for characterization by 16S rRNA gene amplicon sequencing using primers 27f (5′-AGAGTTTGATCCTGGCTCAG-3′) and 1492r (5′-GGTTACCTTGTTACGACTT-3′; Lane 1991), and for which primer 1492r was used as the sequencing primer. DNA sequences were aligned with those from closely related type strains of *Arthrobacter* (Genbank accession numbers AB279889, AB279890, AF102267, AJ512504, X83405, X83406, X80741, X80743, and X83408) over a length of 602 nt in MegAlign (Lasergene; DNAstar, Madison, WI) using the Clustal W algorithm. *Rhodococcus pyridinivorans* (AF173005) was used as an outlier. TreeView (Page 1996) was used to display the phylogram. Unique sequences were deposited in GenBank under accession numbers JN944570–JN944572.

### Growth in liquid medium

Three selected phyllosphere isolates, cp10, cp12, and cp15, were compared with *Arthrobacter chlorophenolicus* A6 (DSMZ culture collection, Braunschweig, Germany; Westerberg et al. 2000) for growth in MM supplemented with 1 mmol/L 4-CP as the sole source of carbon and energy. Growth was followed by measuring the optical density at 600 nm (OD_600_) as a function of time. The experiment was performed in triplicate.

### Phyllosphere performance test

Spontaneous rifampicin-resistant mutants of cp10, cp12, cp15, and A6 were selected by plating on Luria broth (LB) medium with 15 g agar and 20 mg rifampicin per liter. These derivatives and the Rif-resistant model phyllosphere colonizer *Pantoea agglomerans* (synonym: *Erwinia herbicola*) 299R (Brandl et al. 1996) were grown to mid-exponential phase in LB with 20 mg rifampicin per liter at 28°C and 250 rpm and diluted in sterile demineralized water to obtain bacterial suspensions of approximately 1.7 × 10^4^ colony forming units (CFUs)/mL. Two-week-old bean plants (*Phaseolus vulgaris*, green snap bean, variety Blue Lake Bush 274) with the first two leaves fully expanded were dipped into the bacterial suspension. The plants were then incubated for 1 day at 97% air humidity in a closed box in a growth chamber, followed by 1 day at 50% air humidity in an open box and one more day back at 97% air humidity. The growth chamber was set to maintain a day–night cycle of 16 and 8 h at 21 and 16°C, respectively. Growth and survival of bacteria on the foliage was monitored by sacrificing four leaves for analysis at each time point. Bacteria were recovered from individual leaves in 20 mL PBS by 5-sec vortexing, 7-min sonication, and 5-sec vortexing. Dilutions were spread on LB plates containing rifampicin, and CFUs were counted and normalized per gram of leaf tissue.

In a second test, phyllosphere performance of strain cp15 was compared to that of *P. agglomerans* 299R, either inoculated separately or mixed in a 1:1 ratio. Bacterial suspensions used for dipping the bean leaves contained approximately 3.3 × 10^6^ CFU/mL of each strain. The inoculation densities were 100-fold higher than in previous experiment in order to ensure interaction between the two strains. On LB agar plates, both strains could easily be distinguished by morphology.

### 4-Chlorophenol-degradation genes

Primer sets were designed to target homologues of three genes in the *A. chlorophenolicus* A6 4-CP-degradation cluster, namely Achl_4569 (*cphA-I*), Achl_4573 (*cphC-I*), and Achl_4564 (*cphC-II*). These genes encode for one hydroxyquinol 1,2-dioxygenase and two monooxygenase enzymes (Nordin et al. 2005; Unell et al. 2009). Homologous sequences were obtained from GenBank, aligned using MegAlign (Lasergene; DNAstar) and used to design degenerate primers in conserved regions (Table 1). Genomic DNA from strains cp10, cp12, and cp15 was isolated using the ZR Fungal/Bacterial DNA MiniPrep (Zymo Research, Irvine, CA) after prior incubation for 30 min in TE buffer (30 mmol/L Tris-Cl, 1 mmol/L EDTA, pH 8.0) containing 15 mg of lysozyme and 2 mg of proteinase K per mL. PCR mixtures contained 1 U FastStart Taq DNA polymerase, 1× buffer (Roche Diagnostics, Mannheim, Germany), 0.2 mmol/L of each deoxynucleoside triphosphate, 3 μmol/L of each primer, and 5 ng of genomic DNA in a total volume of 25 μL. The PCR cycling regime was (1) one cycle of 2 min at 95°C, (2) 35 cycles of 30 sec at 95°C, 30 sec at 50°C, and 60 sec at 72°C, and (3) one final extension cycle of 10 min at 72°C. PCR products were verified by agarose gel electrophoresis and purified using a Qiaquick PCR purification kit (Qiagen, Venlo, The Netherlands). The fragments were sequenced (Macrogen, Seoul, Korea) from both directions with the same primers used for PCR amplification. Sequences were deposited in GenBank under accession numbers JN944561–JN944563 and JN944566–JN944569.

**Table 1:** Degenerate primers used to amplify *Arthrobacter* genes involved in 4-chlorophenol degradation

Gene	Forward primer (5′-3′)	Reverse primer (5′-3′)	Size in A6 (bp)	NCBI entries used for alignment
*cphA-I*	CARYTNATGCARGCNYTNAC	CRTCYTCRTCNGCYTCCCA	385	YP_002478496; ABL75139; ACX85436; BAI53128
*cphC-I*	ATGAAYGTNGTNATGTTYAC	GRTACCAYTTNGCRTGNTC	538	YP_002478500; ABL75143; ACX85440; BAI53124; BAI53132
*cphC-II*	GCNCAYATHACNAAYCARMG	GCYTTRAANGTDATRTTCAT	485	YP_002478491; YP_831411

## Results and Discussion

**Figure 1:**
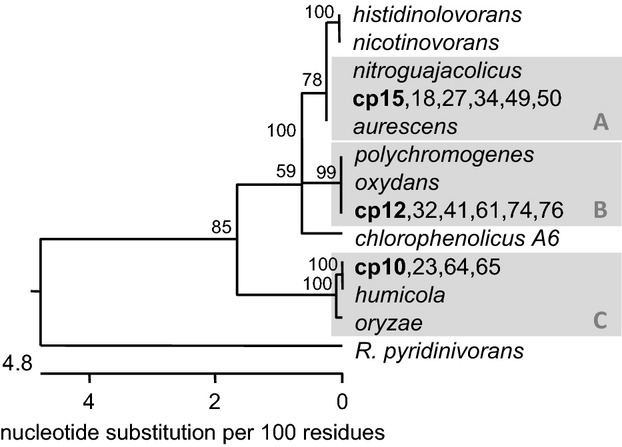
Phylogram based on the alignment of partial 16S rRNA gene sequences of cp isolates and closely related type strains of *Arthrobacter*. The sequence of *Rhodococcus pyridinivorans* was used as outlier. Highlighted boxes indicate three groups (A, B, C) of identical cp sequences. Numbers at nodes indicate % bootstrap values (*n* = 1000 trials).

Leaf surface washes from trees and ground vegetation at six plots in an experimental apple orchard were used to seed two sets of enrichment cultures, one with 4-CP and one with triadimenol. Turbidity representing bacterial growth was observed only in the six 4-CP enrichment cultures that were inoculated with bacteria from ground vegetation. Analysis of the supernatants of these cultures by HPLC showed that 4-CP concentrations had fallen below 10 μmol/L within 2 weeks. At this time, enrichments were spread on 1/10 TSA plates and for each one of the six cultures 12–16 bacterial colonies were selected. For more than half of the isolates, we confirmed the ability to grow on MM containing 1 mmol/L 4-CP as the sole source of carbon and energy (Table 2). Of these 4-CP degraders (“cp isolates”), approximately 25% featured a yellow colony type, while the others were white. Sixteen of the cp isolates were selected for characterization by 16S rRNA gene amplicon sequencing and all were identified as *Arthrobacter* species, belonging to one of three groups (A, B, or C), based on alignment to the 16S rRNA gene sequences of known type strains of *Arthrobacter* species (Fig. 1). Members of group A showed 100% sequence similarity to 4-nitroguaiacol degrader *Arthrobacter nitroguajacolicus*^T^ (Kotouckova et al. 2004) and atrazine-degrader *Arthrobacter aurescens* TC1 (Strong et al. 2002; Mongodin et al. 2006), both soil isolates. All strains with the yellow colony phenotype belonged to this group A. Sequences in group B were identical to those of *Arthrobacter polychromogenes*^T^ (Schippers-Lammertse et al. 1963) and *Arthrobacter oxydans*, both of which were isolated from air, whereas sequences in group C were identical to that of *Arthrobacter humicola*^T^, which was recovered from paddy soil (Kageyama et al. 2008).

**Table 2:** Bacterial isolates obtained from leaf wash enrichment cultures on 4-chlorophenol (4-CP)

	Growth on 4-CP		No growth on 4-CP
		
	Yellow colonies	White colonies	Various morphologies
Plot A	2 (cp15,18)^1^	8 (cp10,12)	2
Plot B	1 (cp27)	6 (cp23,32)	5
Plot C	5 (cp34,49)	4 (cp41)	7
Plot D	2 (cp50)	7 (cp61)	3
Plot E	0	3 (cp64,65)	9
Plot F	0	6 (cp74,76)	7

1 In parentheses are shown the isolates that were selected for characterization by sequencing of the 16S rRNA gene (Fig. 1).

**Figure 2:**
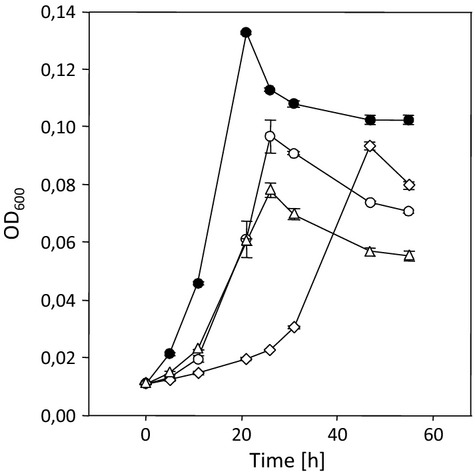
Growth of phyllosphere isolates cp10 (open circles), cp12 (open diamonds), and cp15 (open triangles), compared to that of the 4-chlorophenol (4-CP) model degrader *Arthrobacter chlorophenolicus* A6 (closed circles) on mineral medium supplemented with 1 mmol/L 4-CP as the sole source of carbon and energy. Error bars represent standard deviations, *n* = 3.

From each group, one representative was selected for further characterization: cp15 representing group A, cp12 from B, and cp10 from group C. Figure 2 shows the growth of these isolates on MM with 1 mmol/L 4-CP, compared to that of 4-CP model degrader *A. chlorophenolicus* A6 (DSMZ culture collection, Braunschweig, Germany; Westerberg et al. 2000). *Arthrobacter chlorophenolicus* A6 was the fastest growing strain in this medium and cp12 the slowest. The maximum specific growth rates (μ_max_) for A6, cp10, cp12, and cp15 were 0.118, 0.099, 0.062, and 0.089/h, respectively. In addition, we confirmed by PCR analysis that cp10, cp12, and cp15 carried orthologs of *cphA-I* and *cphC-I*. These genes encode an intradiol dioxygenase and a monooxygenase, respectively, alleged to be involved in 4-CP degradation by *A. chlorophenolicus* A6 (Nordin et al. 2005). PCR for the *cphC-II* gene, encoding another monooxygenase, was positive only for cp12 (Table 3).

**Table 3:** Partial gene fragments of *cphA-I*, *cphC-I*, and *cphC-II* orthologs amplified by PCR from cp isolates

Isolate	Gene	Closest match in GenBank	Species	% Identity
cp10	*cphA-I*	AB530681 (2769..2424)	*Arthrobacter* sp. IF1	100
cp10	*cphC-I*	AB530680 (1555..2053)	*Arthrobacter* sp. IF1	100
cp12	*cphA-I*	CP001343 (81639..81294)	*Arthrobacter chlorophenolicus* A6	80
cp12	*cphC-I*	CP001343 (86948..87446)	*A. chlorophenolicus* A6	85
cp12	*cphC-II*	CP001343 (75047..75491)	*A. chlorophenolicus* A6	76
cp15	*cphA-I*	AB530681 (2769..2424)	*Arthrobacter* sp. IF1	100
cp15	*cphC-I*	AB530681 (8077..8575)	*Arthrobacter* sp. IF1	100

These results confirmed that enrichment on 4-CP was very effective in selectively recovering *Arthrobacter* species from leaf surfaces, at least from ground vegetation in this apple orchard. Prior to the collection of leaf material, the orchard had received weekly applications of the fungicide Exact®, which has triadimenol as an active ingredient. We never found bacterial degraders of triadimenol in enrichment cultures supplemented with triadimenol as sole source of carbon and energy. Under the influence of sunlight, triadimenol can be degraded to 1,2,4-triazole with the release of 4-CP (Iesce et al. 2003; Da Silva and Vieira Ferreira 2004). We do not know to what extent the treatment with triadimenol allowed for the recovery of 4-CP degraders in our study. However, we were never able to recover such degraders from enrichment cultures that were seeded with apple leaves from the same plots. We suspect that the recovery of 4-CP-degrading *Arthrobacter* strains from ground vegetation but not apple leaves was due to the proximity of the former to soil.

**Figure 3:**
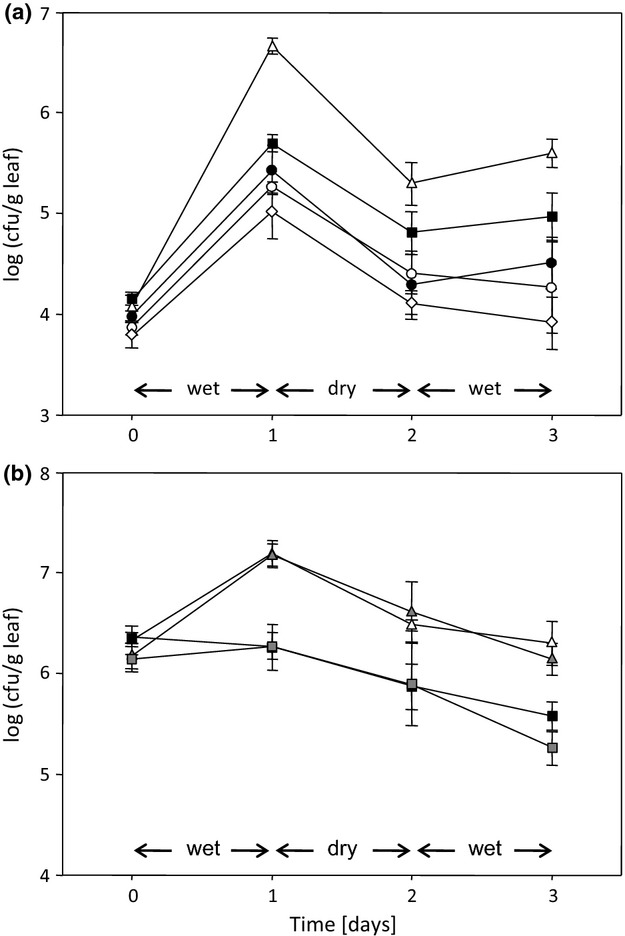
(a) Phyllosphere performance of *Arthrobacter* isolates cp10 (open circles), cp12 (open diamond), and cp15 (open triangles), compared to that of soil bacterium *Arthrobacter chlorophenolicus* A6 (closed circles), and phyllosphere bacterium *Pantoea agglomerans* 299R (closed squares). (b) Phyllosphere performance of *Arthrobacter* isolate cp15 (triangles) and *P. agglomerans* 299R (squares) at high densities, inoculated either alone (cp15 = open symbols; 299R = closed symbols) or in competition with each other (gray symbols). The graphs show bacterial numbers on bean leaves after 1 day at 97% relative humidity (“wet”), 1 day at 50% relative humidity (“dry”), and another day at 97% relative humidity (“wet”). Error bars represent standard deviations, *n* = 4.

To determine whether our cp isolates represented mere “soil contaminants,” that is transients, or whether they were actually capable of growing epiphytically, we compared isolates cp10, cp12, cp15, and *A. chlorophenolicus* A6 to a model bacterium for phyllosphere colonization, that is *P. agglomerans* 299R, in a standard “wet-dry-wet” phyllosphere competency test (Lindow 1993) on bean plants. The results are shown in Figure 3a. Overall, the phyllosphere performance of the four *Arthrobacter* strains resembled that of *P. agglomerans* 299R. In all cases, population sizes had increased at least one order of magnitude after 24 h under conditions of high relative humidity, suggesting that these strains were able to access and utilize nutrients on the leaf surface for growth. All strains showed the expected reduction in population size upon exposure to reduced humidity, while *P. agglomerans* 299R and two of the *Arthrobacter* isolates (cp 15 and A6) recovered from this stress by increasing population sizes in the subsequent 24-h wet period. Strain cp15 appeared to outperform all other strains, including *P. agglomerans* 299R, especially in the first 24-h period. In an additional experiment, phyllosphere performance of cp15 was compared to that of *P. agglomerans* 299R, alone or in competition at high densities (Fig. 3b). Again, the cp15 strain reached higher numbers than 299R, even when they were inoculated together on the same leaf. For both strains, inoculation together with the other strain did not impact the bacterial growth pattern compared to inoculation alone (Fig. 3b).

We conclude then that the *Arthrobacter* strains that were isolated from orchard ground vegetation were good phyllosphere colonizers. The same was true for strain A6, which was originally recovered from soil. It is likely that many of the traits that generally make *Arthrobacter* species excellent survivors in soil (Mongodin et al. 2006), for example desiccation tolerance (Labeda et al. 1976), contributed to the ability of A6 and the cp isolates to deal with and rebound from the imposed stress of low relative humidity on leaf surfaces (Fig. 3). Remarkable is our finding that strains of *Arthrobacter* excelled at reproducing on leaf surfaces under growth-conducive conditions. It is unlikely that this ability depended on the capacity to catabolize 4-CP. In culture, isolate cp15 had the lowest yield on 4-CP, relative to other *Arthrobacter* strains (Fig. 2), yet it showed superior epiphytic growth during the first 24 h on bean leaves. Also, we were not able to recover 4-CP from the surface of bean leaves at concentrations that were detectable by GC-MS (not shown) or that would allow population increases such as those that were seen on leaves for cp10, cp12, and cp15 during the first 24 h on the bean leaf surface (Fig. 3). Thus, foliar growth by *Arthrobacter* under these conditions must be attributed to the acquisition of other carbon and energy sources on the leaf surface, most likely photosynthates such as fructose, glucose, and sucrose, which are among the most abundant sources of carbon on leaf surfaces (Leveau 2006). This does not preclude the possibility that on other plant species, in particular those that are known to harbor phenolic compounds on their leaf surfaces (Yadav et al. 2005), *Arthrobacter* would benefit from the possession of *cph* genes and the ability to catabolize substituted phenols. Moreover, as it is likely that phyllosphere *Arthrobacter* bacteria spend part of their life cycle in the soil environment, carrying *cph* genes might be advantageous for degradation of other aromatic compounds in soil, for example those formed during degradation of lignin and humic acids.

In conclusion, our findings show that members of the genus *Arthrobacter* fit the definition of residual epiphytes and that the presence of *Arthrobacter* on leaf surfaces should be interpreted in light of the demonstrated capacity to reproduce epiphytically. This capacity, together with the notions that *Arthrobacter* species (1) exhibit high levels of resistance to desiccation stress (Labeda et al. 1976), (2) have a wide range of pollutant degradation capabilities (Alvarez and Vogel 1991; Keuth and Rehm 1991; Westerberg et al. 2000; Kotouckova et al. 2004), and (3) can be retrieved as culturable bacteria from leaf surfaces (this study; Ercolani 1991; Thompson et al. 1995; Heuer and Smalla 1999; Krimm et al. 2005; Jackson et al. 2006), makes the *Arthrobacter* genus a promising group for further development as a model for the study of phyllosphere-based bioremediation (Sandhu et al. 2007). Using other bacterial species, such phylloremediation has been demonstrated for a number of pollutants such as toluene, phenol, and phenanthrene (De Kempeneer et al. 2004; Sandhu et al. 2007, 2009; Waight et al. 2007; Yutthammo et al. 2010), as well as for agrochemicals such as dichlorvos and acetamiprid (Ning et al. 2010; Zhou et al. 2011). Phyllosphere isolates of *Arthrobacter* strains may have practical utility as foliar sprays for the initial or accelerated attrition of pesticide residue associated with the use of atrazine, cyanazine, phenylurea herbicides, glyphosate, and malathion, all of which have been reported to be targets for destruction by *Arthrobacter* (Kertesz et al. 1994; Strong et al. 2002; Tixier et al. 2002). Application of biodegradation-capable, phyllosphere-competent strains of *Arthrobacter* to ground cover or buffer-zone vegetation may be a sustainable strategy to mitigate and reduce levels of environmental contamination associated with runoff of pesticides (Reichenberger et al. 2007).
